# Study of the Structure and Mechanical Properties of Ti-38Zr-11Nb Alloy

**DOI:** 10.3390/jfb16040126

**Published:** 2025-04-02

**Authors:** Konstantin V. Sergienko, Sergei V. Konushkin, Yaroslava A. Morozova, Mikhail A. Kaplan, Artem D. Gorbenko, Boris A. Rumyantsev, Mikhail E. Prutskov, Evgeny E. Baranov, Elena O. Nasakina, Tatiana M. Sevostyanova, Sofia A. Mikhlik, Andrey P. Chizhikov, Lyudmila A. Shatova, Aleksandr V. Simakin, Ilya V. Baimler, Maria A. Sudarchikova, Mikhail L. Kheifetz, Alexey G. Kolmakov, Mikhail A. Sevostyanov

**Affiliations:** 1 A.A. Baikov Institute of Metallurgy and Materials Science, Russian Academy of Sciences (IMET RAS), 119334 Moscow, Russia; venev.55@mail.ru (S.V.K.); yasya12987@gmail.com (Y.A.M.); mishakaplan@yandex.ru (M.A.K.); artemgorbenk@yandex.ru (A.D.G.); brumyantsev@imet.ac.ru (B.A.R.); mprutskov@imet.ac.ru (M.E.P.); ebaranov@imet.ac.ru (E.E.B.); nacakina@mail.ru (E.O.N.); beliyfecity@yandex.ru (S.A.M.); mariahsudar@yandex.ru (M.A.S.); akolmakov@imet.ac.ru (A.G.K.); cmakp@mail.ru (M.A.S.); 2 National Medical and Surgical Center Named after N.I. Pirogov of the Ministry of Health of the Russian Federation, 117513 Moscow, Russia; tata_sev1048@mail.ru; 3 Moscow Regional Research and Clinical Institute (“MONIKI”), 129110 Moscow, Russia; 4 Institute of Structural Macrokinetics and Materials Science, Russian Academy of Sciences (ISMAN), 142432 Chernogolovka, Russia; 5 Faculty of Radio Engineering and Electronics, Voronezh State Technical University, 394026 Voronezh, Russia; lashatova1@mail.ru; 6 Prokhorov General Physics Institute of the Russian Academy of Sciences, Vavilov Str. 38, 119991 Moscow, Russia; avsimakin@gmail.com (A.V.S.); ilyabaymler@yandex.ru (I.V.B.); 7 Institute of Applied Physics, NAS of Belarus, 220072 Minsk, Belarus; kheifetz@iaph.bas-net.by; 8 Federal State Budgetary Scientific Establishment the All-Russian Scientific Research Institute of a Phytopathology, 143050 Bolshye Vyazemy, Russia

**Keywords:** titanium alloys, β-titanium, medical materials, mechanical properties

## Abstract

Hip joint implants are among the most prevalent types of medical implants utilized for the replacement of damaged joints. The utilization of modern implant materials, such as cobalt–chromium alloys, stainless steel, titanium, and other titanium alloys, is accompanied by challenges, including the toxicity of certain elements (e.g., aluminum, vanadium, nickel) and excessive Young’s modulus, which adversely impact biomechanical compatibility. A mismatch between the stiffness of the implant material and the bone tissue, known as stress shielding, can lead to adverse outcomes such as bone resorption and implant loosening. Recent studies have shifted the focus to β-titanium alloys due to their exceptional biocompatibility, corrosion resistance, and low Young’s modulus, which is close to the Young’s modulus of bone tissue (10–30 GPa). In this study, the microstructure, mechanical properties, and phase stability of the Ti-38Zr-11Nb alloy were investigated. Energy dispersion spectrometry was employed to confirm the homogeneous distribution of Ti, Zr, and Nb in the alloy. A subsequent microstructural analysis revealed the presence of elongated β-grains subsequent to rolling and quenching. Furthermore, grinding contributed to the process of recrystallization and the formation of subgrains. X-ray diffraction analysis confirmed the presence of a stable β-phase under any heat treatment conditions, which can be explained by the use of Nb as a β-stabilizer and Zr as a neutral element with a weak β-stabilizing effect in the presence of other β-stabilizers. Furthermore, the modulus of elasticity, as determined by tensile testing, exhibited a decline from 85 GPa to 81 GPa after annealing. Mechanical tests demonstrated a substantial enhancement in tensile strength (from 529 MPa to 628 MPa) concurrent with a 32% reduction in elongation to fracture of the samples. These alterations are attributed to microstructural transformations, including the formation of subgrains and the rearrangement of dislocations. This study’s findings suggest that the Ti-38Zr-11Nb alloy has potential as a material of choice due to its lower Young’s modulus compared to traditional materials and its stable β-phase, which enhances the implant’s durability and reduces the risk of brittle phases forming over time. This study demonstrates that the corrosion resistance of titanium grade 2 and Ti-38Zr-11Nb is comparable. The material in question exhibited no evidence of cytotoxic activity in the context of mammalian cells.

## 1. Introduction

The hip joint is a critical component of the human musculoskeletal system, subject to significant stress [1]. In cases of degenerative joint diseases or substantial joint damage resulting from injuries, artificial hip joint implantation may be indicated to restore functionality. The fabrication of the leg and cup of the hip joint necessitates the utilization of materials that exhibit a set of properties, including biocompatibility, sufficient mechanical properties, corrosion resistance, and a Young’s modulus close to the value of the bone index. The annual increase in the number of such surgical interventions is associated with several factors, including an aging population, increased accessibility of surgical procedures in various regions worldwide, heightened awareness of the potential benefits of artificial joint replacement, and a rise in the success rate of operations [2,3]. The materials utilized in contemporary hip arthroplasty can be categorized into the following groups: cobalt–chromium alloys, stainless steel, titanium, and titanium alloys [4,5,6].

Despite their high strength and corrosion resistance, some of these materials contain elements that are toxic to the human body, such as aluminum, vanadium, and nickel [7,8,9,10]. Furthermore, their elastic modulus (Young’s modulus) is much higher than that of bone tissue [11,12], which can negatively affect biomechanical compatibility due to the risk of aseptic loosening, wear, and mismatch of the mechanical properties of the implant with those of the bone tissue [13].

The modulus of elasticity (E) is a material property that governs its stiffness and resistance to deformation under tension or compression. For materials utilized in endoprosthetics, it is imperative that the modulus of elasticity approximates that of bone tissue, which ranges from 10 to 30 gigapascals (GPa) [14,15]. This match ensures optimal mechanical biocompatibility between the implant and the surrounding bone tissue, which promotes uniform load distribution and minimizes the risk of localized over-stress in the bone–implant system [16,17,18,19,20].

A phenomenon referred to as stress shielding occurs when the Young’s modulus of the implant material is significantly higher than that of the bone. Consequently, the load is distributed unevenly between the implant leg and the bone into which it is integrated. Consequently, a portion of the bone tissue experiences an atypically low local load, thereby initiating a process that surpasses the rate of bone formation by that of bone resorption. Cobalt–chromium alloys, which possess high strength and wear resistance, exhibit high stiffness (Young’s modulus ~134–230 GPa) [21,22]. Stainless steel, on the other hand, is less frequently utilized due to its diminished biocompatibility and corrosion resistance, and its Young’s modulus is elevated at ~190–210 GPa [23,24], which also surpasses the Young’s modulus of bone tissue (10–30 GPa). Titanium and titanium alloys are extensively utilized due to their exceptional biocompatibility, corrosion resistance, and moderate Young’s modulus (~100–110 GPa for Ti-6Al-4V). However, pure titanium and the majority of its alloys (with α, α + β phase) still possess an excessive Young’s modulus relative to bone tissue, which can result in uneven load distribution. In accordance with Wolff’s Law [25], bone adapts to the applied loads: insufficient mechanical stimulation activates bone resorption processes, leading to the gradual thinning and weakening of the bone. This phenomenon can result in the implant’s fixation being compromised, consequently leading to the further loosening of the implant. This, in turn, necessitates a secondary surgical intervention for the replacement of the implant leg.

In relation to the aforementioned points, the examination of materials exhibiting a reduced Young’s modulus in comparison to the utilized materials has the potential to diminish the progression of bone destruction over time. This, in turn, may result in an extended operational lifespan of the product.

For an extended period, β titanium alloys did not garner the attention of industry; however, recent research and a re-evaluation of the applicability of materials found in them have identified a promising replacement for the materials currently in use. This is due to a successful combination of properties, including high biocompatibility, adequate corrosion resistance, and, most significantly, a lower Young’s modulus relative to Ti–6Al-4V and cobalt–chromium alloys. The development of β-titanium alloys with a Young’s modulus of approximately 50–80 GPa facilitates the creation of bone implants with a more natural stiffness, thereby minimizing the risk of stress shielding and further bone resorption [26]. Furthermore, the optimization of phase composition, characterized by the predominance of β-phase, and the alloying with biocompatible elements, such as Zr and Nb, facilitate the elimination of toxic components and enhance osseointegration [27,28,29,30,31]. In the future, it will be possible to use these advantages to create implantable products that can ensure uniform load distribution and reduce the frequency of repeated operations. Nevertheless, further research is necessary to optimize the composition of these alloys, assess their fatigue resistance, and ascertain their long-term stability in biological environments before their introduction into clinical practice can be considered. The present study contributes to these challenges by focusing on analyzing the relationship between the structure, phase composition, and mechanical properties of β-titanium alloys, which is a critical step towards their practical application in endoprosthetics.

The aim of this study is to systematically evaluate a beta-titanium alloy that has the potential to become a new material for medical use. In this study, the effect of annealing on the phase composition and complexity of mechanical properties was evaluated. Attention was paid to the study of the elastic properties of the alloy, a key factor in the effectiveness of the integration of alloys with human bone. This is paramount for ensuring the longevity and efficacy of the implants. The present study encompassed a comprehensive evaluation of the physical and strength characteristics of the alloy.

## 2. Methods and Materials

### 2.1. The Object of Research

The object of research is alloy (at.%) Ti-38Zr-11Nb. The titanium was manufactured by PJSC VSMPO-AVISMA (Moscow, Russia) and the alloying elements were manufactured by Chepetsky Mechanical Plant Stock Company (Glazov, Russia). For a preliminary assessment of the phase composition, the molybdenum equivalent method was used, which allows us to calculate the required amount of alloying elements and their composition to obtain the required phase [32].

Alloys VT1-0 (An analog of Titanium Grade 2 alloy) and VT6 (An analog of Titanium Grade 5 alloy) were the object of comparison.

To perform the calculation, it is necessary to convert atomic percentages to mass percentages (Table 1).

To calculate the molybdenum equivalent in a titanium alloy with several alloying elements, it is necessary to add up the molybdenum equivalent values for each alloying element. At the same time, the influence of α-stabilizers is neglected. Each alloying element has an individual β-phase stabilization force. Thus, it is known that niobium has a 3.6-fold effect on β phases in relation to molybdenum. Zirconium is usually not a beta stabilizer for titanium; however, when other elements that are beta stabilizers are present in the alloy, zirconium also acts as a beta stabilizer and its beta phase stabilization force is 14 times less than that of molybdenum. Each alloying element has an individual β-phase stabilization force. Niobium has a 3.6-fold lower effect on β phases in relation to molybdenum. Zirconium is usually not a beta stabilizer for titanium; however, when other elements that are beta stabilizers are present in the alloy, zirconium also acts as a beta stabilizer and its beta phase stabilization force is 14 times less than that of molybdenum [32,33].

Calculation of the molybdenum equivalent:

[Mo] eq = %Nb × 0.28 + %Zr × 0.08 = 15 × 0.28 + 51 × 0.08 = 8.28

The obtained value of the molybdenum equivalent refers the alloy under study to (α + β)-titanium alloy (Table 2). Using quenching, it is possible to obtain an unstable β phase, which differs from the (α + β) phase by a lower modulus of elasticity. Using quenching, it is possible to obtain an unstable β phase, which differs from the (α + β) phase by a lower modulus of elasticity.

### 2.2. Production and Preparation of the Material

The ingots were smelted in a vacuum argon arc furnace with a non-consumable electrode. After the furnace (L200DI (Leybold-Heraeus, Hanau, Germany)) was evacuated to 1.33 Pa, argon was supplied to the furnace up to a pressure of 40 × 10^3^ Pa. The sequence of metals in the arrangement was dictated by the respective melting temperatures of the materials, with the melting process being initiated by an arc that propagated from the top downward. Before melting the ingots of the studied materials, a getter was initially melted, which is located in a separate well. This is required for additional argon purification in the furnace. The process of remelting was repeated seven times, with the ingots undergoing a process of turning over to ensure adequate mixing of the raw materials. The implementation of these remelting operations was meticulously executed to ensure the uniformity of the chemical composition throughout the entirety of the ingot volume. The duration of each ingot melting was 1–1.5 min. After melting, the ingot had the shape of a “boat” with a length of ~120 mm, width of ~25 mm, and height of ~12 mm.

Due to the different melting points of the materials used, the alloy contains dendrites after smelting. Annealing in a vacuum furnace ESKVE-1.7.2.5/21 ShM13 (NITTIN, Moscow, Russia) was used to equalize the alloy composition. The applied mode was at 1000 °C for 4 h. To prevent oxidation, the furnace maintained a vacuum environment with a pressure of 27 × 10^−4^ Pa.

Thermomechanical processing was carried out by the method of multi-stage hot-rolling at a temperature of 700 °C. The resulting plate, 1 mm thick, was quenched at a temperature of 600 °C. Heating at this temperature was conducted for 5 min. The samples from the plate were made using DK7745 electro-erosion treatment (Meatec, Moscow, Russia).

Polishing of the samples was carried out on a Phoenix 4000 machine (Buehler, Lake Bluff, IL, USA). For optical studies, the material was treated with a solution of 10% HF: 10% HNO_3_: 80% H_2_O.

### 2.3. Methods of Research

The determination of oxygen and nitrogen impurities was achieved through a reduction melting process in a graphite crucible within a helium-fueled pulse-resistance furnace, employing the TC-600 analyzer (LECO, 3000 Lakeview Avenue, St. Joseph, MI, USA). Nitrogen was detected using thermal conductivity, and oxygen was detected by the amount of released CO_2_ using the infrared absorption method.

To determine hydrogen impurities, we used reduced melting in a graphite crucible in an argon-fueled pulse-resistance furnace using the RHEN-602 analyzer (LECO, 3000 Lakeview Avenue, St. Joseph, MI, USA). Hydrogen was detected using thermal conductivity.

To determine carbon and sulfur impurities, we used oxidizing melting in a ceramic crucible in an induction furnace with the flux using a CS-600 analyzer (LECO, 3000 Lakeview Avenue, St. Joseph, MI, USA). The elements were detected by the amount of released CO_2_ and SO_2_ using the infrared absorption method. The distribution of elements was studied using a scanning electron microscope (SEM) EM6900 (KYKY, Beijing, China) with an EDS detector from Oxford Instruments (Oxford Instruments, Abingdon, Oxfordshire, UK) using Aztec (version 6.0 SP2) software.

X-ray phase analysis was performed on the DX2700mini diffractometer (Dandong Haoyuan Instrument Co., Dandong, China). The studies were carried out in CuKα = 1.54178 Å radiation, 2θ = 20–100° range; the shooting step was 0.02°/s, the holding time per step was 0.2 s, the voltage on the tube was 40 kV, and the current was 13 mA. The analysis of the obtained diffraction patterns was carried out in the Match! 3 program.

For optical examination of the microstructure, the samples were pre-pressed in a rigid mold into cylinders by means of one-sided pressing.

The samples were subjected to pressure within an IPA 40 air-hydraulic press (Remet, Bologna, Italy) using Aka-Resin Epoxy resin at temperatures ranging from 160 to 185 °C, with a recommended holding time of 20 min. This temperature range is recommended by the manufacturer of the epoxy resin. The sample was then placed into a mold, which was filled with granulated resin. Thereafter, the chamber was closed and the sample was compressed to a pressure of 6 bar. Subsequent to this, the device executed a predetermined program. Thereafter, the compressed sample was subjected to a grinding and polishing procedure. The grinding and polishing of samples for subsequent examination via optical microscopy was carried out in the following sequence.

On a Piatto diamond disk with the following grit:

P220 for 10 min;
P600 for 10 min;
P1000 for 5 min;
P1200 for 5 min;
P2500 for 5 min;
P4000 for 5 min.

The polishing was done on the Akasel NAPAL velvet polishing wheel with the DiaMaxx Poly diamond suspension. The surface of the samples was etched with a solution of nitric and hydrofluoric acids with distilled water in a ratio of HF:HNO_3_:15 H_2_O by volume. The sections were immersed in the etching solution for 5–30 s, then washed with running water.

The microstructure was examined on the microscope MET 5C (Altami, Saint Petersburg, Russia) [34].

Nanohardness and Young’s modulus were determined using a NanoScan-4D nanohardness tester (NauchSpecPribor, Troitsk, Russia). Instrumental indentation was performed according to ISO 14577-1:2002 [35]. All specimens were subjected to instrumental indentation into the matrix with a load of 500 mN using a Berkovich type diamond tip. Measurements were taken across the full width of the specimen. The load/unload rate was 20 mN/s, the contact time was 20 s, and the distance between indentations was 200 μm. The number of indentations for each treatment type was 27. The experimental curves were processed using NANOINDENTATION 3.0 software from CSM Instruments (Peseux, Switzerland) with a specified Poisson’s ratio (0.3) and were averaged over five experimental curves. Elastic recovery was defined as the ratio of elasticity to total indentation work. Fractographic studies were carried out on a KYKY EM6900 (KYKY, Beijing, China) scanning electron microscope using a secondary electron detector at an accelerating voltage of 20 kV.

Mechanical studies were carried out on the Instron 3382 (Instron, Norwood, MA, USA) equipment. The data were processed in the INSTRON Bluehill 2.0 software program According to the standard GOST 1497-84 [36]. The following parameters were determined: conditional yield strength σ_0.2_, tensile strength σ_v_, and relative elongation δ.

For the long-term immersion corrosion tests of Ti-38Zr–11Nb alloy samples, two types of samples were used for immersion tests—polished and with a rough surface—to simulate the surface treatment of the implant and stimulate the release of metal ions, as well as reference samples (VT1-0 and VT6) with periodic determination of the metal release into an environment simulating physiological conditions. Since the material is supposed to be implanted in the hip area, where it will come into contact with synovial (articular) fluid—it is a viscous liquid that fills the joint cavity and plays a key role in maintaining joint health, and it is produced from blood serum, normally pH 7.34–7.43—the tests were carried out in Ringer’s solution (pH 7.34), a standard buffer medium based on 0.9 wt.% NaCl.

Double-distilled water was used in the work. Standard solutions of the elements were prepared from metals with a high degree of purity (99.9%) or from certified standard solutions from Merk. The background is 1 N hydrochloric acid.

Samples of each type were placed in 100 mL of the used medium contained in flat-bottomed thermolab glass flasks. The vials were tightly sealed and stored in the dark between sampling at 37 °C. After the selected period (7, 14, and 21 days) of exposure of samples in the medium, samples were taken from the flasks for analysis (volume—2 mL) and diluted 5 times with 1.25 M HCl medium, which ensures the stability of analytes in solution [37]). The initial buffer solutions were used as reference solutions. The research was carried out using an ICP-OES EXPEC 6500 inductively coupled plasma atomic emission spectrometer (EXPEC Technology, Beijing, China).

The induction argon plasma is the most efficient source of atomic emission, which, in principle, can be used to determine all elements except argon. Induction plasma is suitable for the determination of a wide range of concentrations, from ultra-low to macro-concentrations. NPP with ICP is less susceptible to interference than any other comparable spectrometric method.

The atoms and ions of the sample in the plasma are in an excited state. The intensity of the radiation emitted as the atoms and ions transition to lower energy levels is measured. Each element emits a specific wavelength of radiation. Analysis of the resulting spectrum allows for qualitative and quantitative analysis. The analyte passes through the central channel into the plasma zone and is heated to a temperature of approximately 8000 K. At this temperature, almost complete atomization, a high degree of atomic excitation, and partial ionization are achieved. A zone above a brightly glowing plasma is used to obtain the spectrum. Here, the atomic radiation can be measured at a low background level.

A 0.9% sodium chloride saline solution, widely used in medicine, was selected to evaluate corrosion resistance. The model solution used in the experiments is used to prepare dosage forms for injection, so the evaluation of this chloride concentration for corrosion potential, corrosion current density, and passivation current density is an urgent and practically important task.

To assess the corrosion resistance of Ti-38Zr-11Nb alloy samples, as well as titanium alloys VT6 and VT1-0 commercially produced and used in medical applications, the potentiodynamic method according to GOST 9.912-89 was used [38]. Accelerated testing methods for resistance to pitting corrosion were used. A silver chloride reference electrode was used for the study in a three-electrode cell. The auxiliary electrode was a platinum wire with a surface area 3 times larger than the area of the working electrode. The tests were carried out at room temperature. To prepare the studied titanium alloy plates, round samples with an area of 1 cm^2^ were cut out on a wire erosion machine. Next, a wire was welded to each plate using spot welding to make an electrical contact. At the next stage, the plates were filled with epoxy resin. After curing the epoxy resin, the working surface of the plates was sanded and polished using 4000 grit sandpaper at the finishing stage. A potentiostat, the galvanostat P-30JM (Ellins, Chernogolovka, Russia) was used to construct the polarization curves. The scanning speed in the potentiodynamic mode is 1 mV/s. Considering that titanium alloys are passive, for a complete assessment of the passive zone, the potential sweep was carried out up to 4.5 V.

In vitro biocompatibility studies.

For in vitro testing, the samples were placed in the wells of a 96-well tablet. The samples were sterilized at 180 °C in a frying pan.

The direct contact method was used to study the adhesive characteristics of materials and determine their cytotoxicity for cells. In carrying out this study, we used a primary culture of human mesenchymal stem cells isolated from pulp, which was obtained from an institution licensed by the Ministry of Health of the Russian Federation operating within the framework of the legislation of the Russian Federation. Cell culture was performed in DMEM/F-12 medium (1:1, Life Technologies, Carlsbad, CA, USA), with 10% ETS, 2 mM L-glutamine, 100 U/mL penicillin, and 100 mcg/mL streptomycin vitamin solution (PanEco, Moscow, Russia) at 37 °C in an atmosphere of 5% OF CO_2_. As they grew and reached a subconfluent state, the cells were treated with 0.25% trypsin-EDTA solution and passed into new vials in a ratio of 1:2. Cells on passage 4 were used for research.

The cells were seeded onto the surface in cells of a 96-well tablet at a concentration of 30 thousand cells/cm^2^ (DMEM/F12 + 10% FBS medium). Cover glasses of the same size were used as a control.

After the end of cultivation, the viability of cells cultured on the surface of the studied materials was evaluated using an Axiovert 200 fluorescence microscope. The method of fluorescent staining cells with SYTO 9 and propidium iodide was used for the analysis. The fluorescent dye SYTO 9 in the study mode λvozb = 450–490 nm and λemiss = 515–565 nm stains the DNA and RNA of living and dead cells green. The intercalating reagent propidium iodide in the study mode λvozb = 546 nm and λemiss = 575–640 nm stains the nuclei of dead cells red.

To study the cytotoxicity of the samples, they were studied by direct contact.

The studies were carried out using hoods and the materials themselves in accordance with the requirements of GOST R ISO 10993.5-99, ‘Medical devices. Assessment of the biological effect of medical products. Part 5. Cytotoxicity testing: in vitro methods.’ [39], and GOST R ISO 10993.12-99, ‘Medical devices. Assessment of the biological effect of medical products. Part 12. Sample preparation and standard samples.’ [40].

The study of cell adhesion and growth on the surface of materials was carried out using NCTC clone L929 cells and primary culture of mouse fibroblasts obtained from the skin and muscle tissue of 13-day-old embryos of C57BL/6-Tg(ACTbEGFP)1Osb/J mice. The cell culture in passage 4 was used to test the materials. The cells were cultured at 37 °C in an atmosphere of 5% CO_2_ in DMEM/F12 (1:1) medium with the addition of 5% embryonic veal serum (ETS) and 100 U/mL penicillin/streptomycin.

DMEM/F12 culture medium with the addition of 5% fetal veal serum (ETS) and 100 mg/mL of penicillin/streptomycin was used as a model medium for the preparation of extracts. The extracts were prepared in sterile foam flakes with asepsis for 4 days at 37 °C. The ratio of the surface area of the material in square centimeters and the volume of the model medium in milliliters for the materials allowed us to determine the amount of medium required for incubation (600–800 µL for each type of alloy using all of the samples presented). Three takes were used for sampling and control.

The cytotoxicity of extracts was assessed by adding them to the cell culture in the wells of a 96-well plate (6–8 wells per variety), followed by cultivation at 37 °C for 1 and 3 days. The inoculation concentration of cells was 10,000/cm^2^, the concentration of embryonic veal serum in the culture medium was 5%. As a control, appropriate dilutions of DMEM/F12 medium of 5% ETS were used, aged for 1 and 6 days at 37 °C. The cytotoxicity of the extracts was determined by cell viability relative to the control using the MTT test. An amount of 10% DMSO was used as a positive control, and a culture medium exposed to the conditions and procedures of the study was used as a background control. A day after the start of cultivation, 10 mL of MTT solution was added to each well (5 mg/mL in a PBS solution [150 mM sodium phosphate buffer, pH 7.2, 150 mM NaCl] filtered through filters with a pore diameter of 0.2 microns). After holding for 3 h at 37 °C in a humidified atmosphere of 5% CO_2_, the liquid was removed, 100 µL of dimethyl sulfoxide (DMSO) was added, and the formed formazan salts were dissolved by shaking the plates at room temperature for 5 min. The color development was recorded by measuring the optical density at a wavelength of 540 nm in the wells of a 96-well plate using a photometer (model 680 BIO-RAD, Hercules, CA, USA). The number of parallel experiments was at least three.

Upon completion of cultivation, samples were prepared for examination using scanning electron microscopy (SEM). The samples were washed in a 0.1 M phosphate–salt buffer (pH 7.4) and fixed for 12 h at a temperature of 5 °C in 2.5% buffered glutaraldehyde solution. After fixation, the samples were washed with water and dehydrated at 4 °C sequentially in a battery of an aqueous ethanol solution of increasing concentrations of 50%, 75%, 80%, and 90% and in absolute ethanol at the final stage. At each stage, the samples were immersed twice for 5 min in an appropriate solution of ethyl alcohol. To remove the alcohol, the samples were transferred to hexamethyldisilazane (HMDS) for 30 min, after which they were dried in air. The morphology of cells on the surface of the samples was studied using a KYKY-SEM 6900 scanning electron microscope (KYKY, Beijing, China) in secondary electrons (SE type detector).

## 3. Results

### 3.1. Chemical Analysis of Alloy Plates by Energy-Dispersive Spectrometry

**Figure 1:**
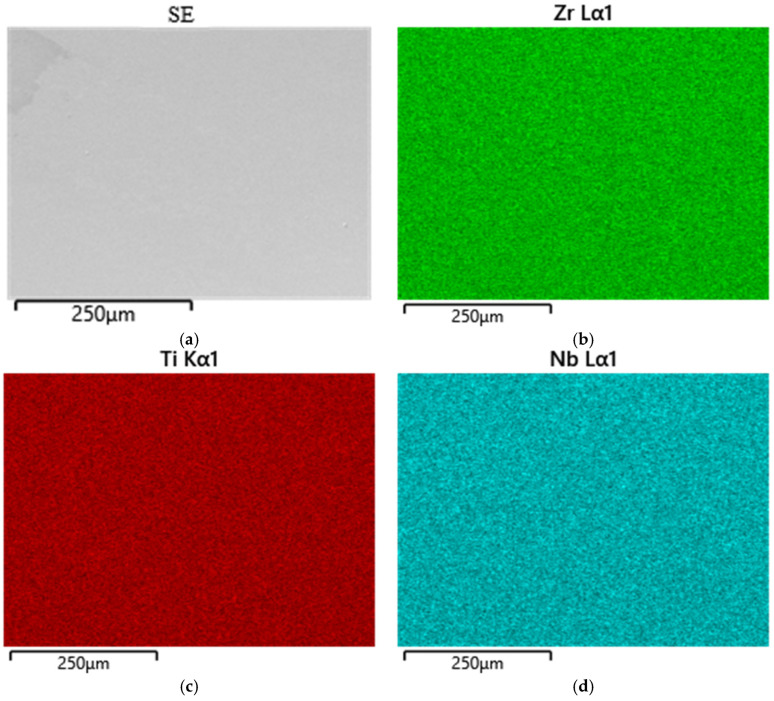
Distribution of elemental composition by area. (**a**) Secondary electrons image, (**b**) distribution of zirconium atoms, (**c**) distribution of titanium atoms, (**d**) distribution of niobium atoms.

The results of the chemical analysis of Ti-38Zr-11Nb alloy plates using energy-dispersive spectrometry show the uniformity of element distribution over the studied area, as well as the result at selected points on the plate surface. The electron image of the studied area and the uniformity of the chemical element distribution, as well as the points of the composition analysis, are shown in Figure 1a–d.

**Figure 2:**
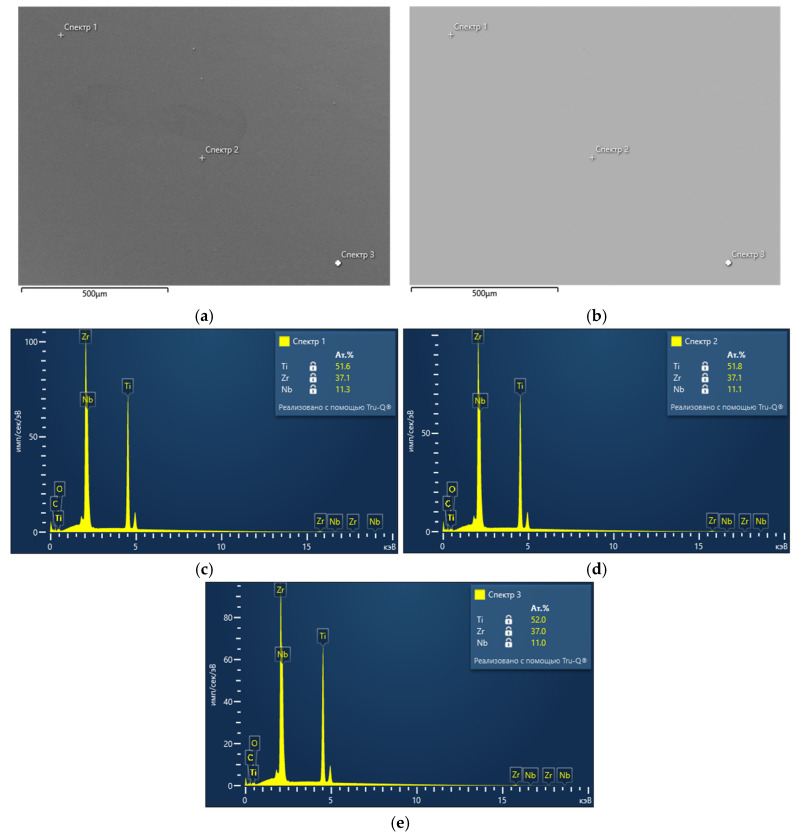
Results of chemical analysis of polished Ti-38Zr-11Nb alloy plate: (**a**) image of SE plate surface; (**b**) image of BSE plate surface; (**c**) results of chemical analysis at point 1; (**d**) results of chemical analysis at point 2; (**e**) results of chemical analysis at point 3.

Confirmed alloy composition: Ti—51.8% (at.), Zr—37.1% (at.), and Nb—11.1% (at.). An example of the spectrum obtained for three points is shown in Figure 2.

Table 3 presents the results of the tests of the alloy’s impurity composition.

The results of the chemical analysis showed that the obtained alloys correspond to GOST 19807-91 [41] for titanium alloys of the VT1-0 type in terms of the amount of impurities.

### 3.2. Optical Microscopy of Microstructures of Ti-38Zr-11Nb Plates

**Figure 3:**
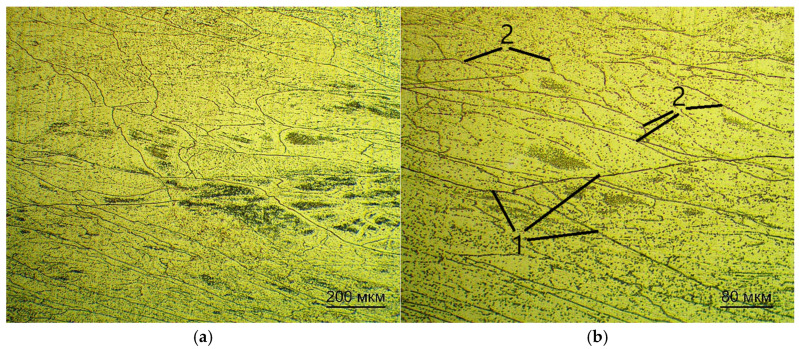
Microstructure of Ti-38Zr-11Nb alloy plates after quenching: 1—the β-grains; 2—the subgrains. (**a**) the scale is 10× (200mkm scale line), (**b**) the scale is 20× (80mkm scale line).

Figure 3 shows the microstructures of the plates after quenching, obtained by studying the cross-section relative to the rolling direction.

**Figure 4:**
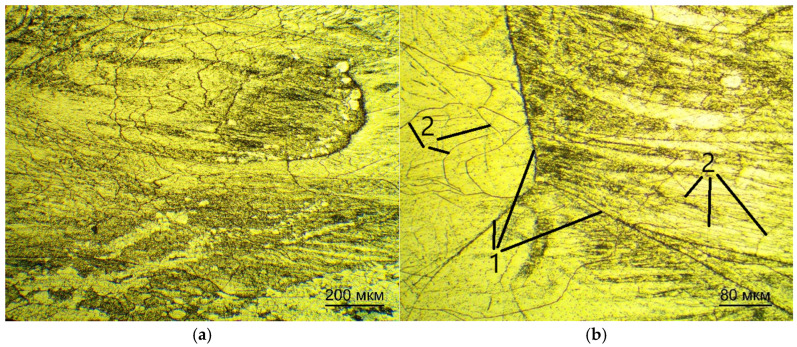
Microstructure of Ti-38Zr-11Nb alloy plates after quenching and annealing: 1—the β-grains; 2—the subgrains. (**a**) the scale is 10× (200mkm scale line), (**b**) the scale is 20× (80mkm scale line).

Figure 4 shows the microstructures of the plates after quenching and annealing at 400 °C for 1 h in a vacuum, obtained by examining the cross-section relative to the rolling direction.

After quenching, the microstructure is elongated along the rolling direction and consists of grains of elongated geometry along the rolling direction. The non-uniformity of the grains can be attributed to two factors. Firstly, ingots and semi-finished products had a low mass in relation to the massive cold rollers of the machine and cooled quickly. Secondly, the time in the heated state was insufficient for recrystallization. Annealing allows for the recrystallization process to occur at a low temperature due to sufficient exposure time and possible excess internal energy in the grains after quenching. Grain growth is associated with the recrystallization process and the formation of subgrains is observed at the same time.

### 3.3. X-Ray Phase Analysis (XRD) of Ti-38Zr-11Nb Plates

The X-ray phase analysis was performed after two types of heat treatment. Only the β phase was detected. This is due to the influence of stabilizing elements [42]. The phase stability after annealing was confirmed. That result confirms that long-term use of the studied material after quenching during aging will not change its phase, and, accordingly, Young’s modulus will not increase to high values.

**Figure 5:**
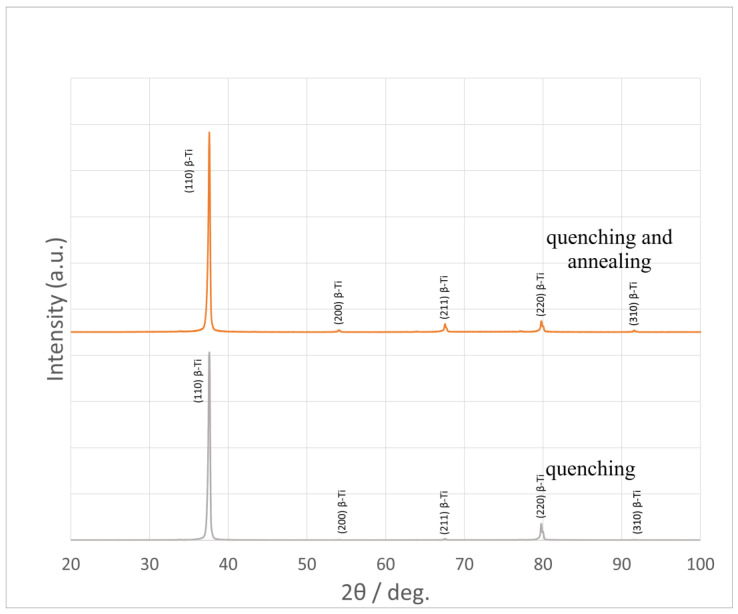
X-ray diffraction patterns of alloys.

From the diffraction patterns in Figure 5, it is evident that the sample of the material with the studied amount of Nb, the formation of α′-Ti, and ω-Ti after quenching is suppressed and completely replaced by β-Ti. The characteristics of the crystal lattice are shown in the Table 4.

The material samples after annealing show peaks in planes (200) and (310). This is due to the reduction in the hardening stresses, the beginning of recrystallization of the grains, and the reduction in the texture obtained after rolling.

### 3.4. Nanohardness of the Ti-38Zr-11Nb Alloy

The nanohardness of Ti-38Zr-11Nb alloy samples in the condition after quenching from 600 °C, as well as after quenching from 600 °C and annealing at 400 °C for 1 h, was determined using the nanoindentation method. The results are presented in Table 5.

The nanohardness of the plates decreases slightly after annealing, associated with a decrease in dislocation density, while the phase composition remains the same. Unlike microhardness, which reflects the average properties at the microstructure level, nanohardness is measured at ultra-low loads (millinewtons) and allows for the analysis of local mechanical properties at the level of individual grains, their boundaries, or nanoscale regions. This makes the nanohardness method highly sensitive to microstructural changes such as recrystallization, subgrain formation, the redistribution of residual stresses, or the appearance of nanoclusters that are not detected using X-ray diffraction.

### 3.5. Mechanical Properties

**Figure 6:**
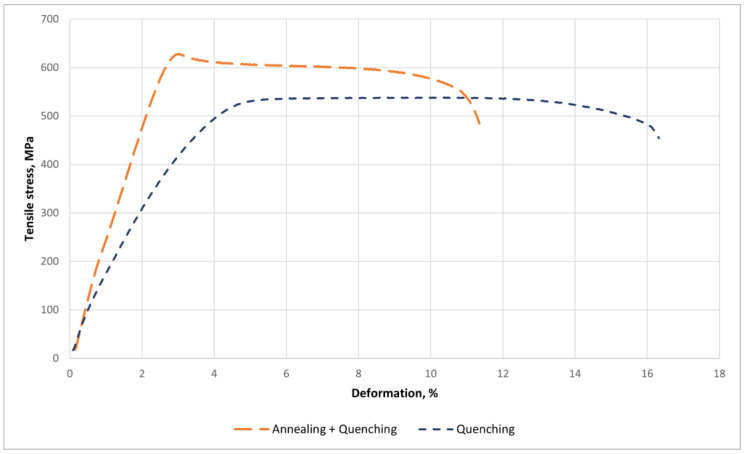
Stress–strain curve of Ti-38Zr-11Nb.

Mechanical test results for the Ti-38Zr-11Nb alloy are shown in Figure 6 and in Table 6.

Yield strength nearly doubled from 371 to 614 MPa. Tensile strength also increased, but not as significantly, from 529 to 628 MPa. The relative elongation decreased by 32%, indicating a decrease in ductility. Based on the XRD data on the preservation of the cubic beta phase and microstructural changes such as grain size, it can be assumed that the presence of subgrains leads to an increase in strength and a decrease in ductility. Residual stresses or defect distribution may also play a role. After quenching, the material may have a high dislocation density and residual stresses. Low-temperature annealing can promote relaxation of these stresses and redistribution of dislocations, possibly forming a more stable structure, such as the transition from an unstable beta phase to a stable one. This can increase yield strength and strength due to dislocation hardening, but reduce ductility due to a decrease in deformability. It is also possible that annealing caused the atoms in the beta phase to become ordered, creating a short-range order that is not detectable using X-ray diffraction, but which affects the mechanical properties. The ordered structure can impede the movement of dislocations, increasing strength, but making the material more brittle [43]. Another factor is the change in grain size and the formation of subgrains observed in optical studies; the formation of a sub-microstructure occurred, which increases strength. It is also worth considering the possible release of very small amounts of other phases that are not detected using X-ray diffraction due to their small size or low concentration, for example, nanoscale clusters or phase nuclei that do not form a crystal lattice distinguishable by X-ray diffraction.

### 3.6. Cyclical Loading

**Figure 7:**
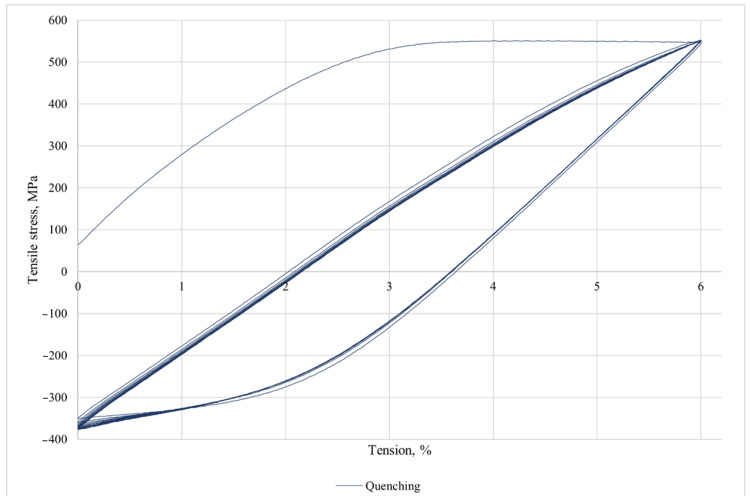
Cyclic stress–strain curve of the Ti-38Zr-11Nb.

Figure 7 shows the stress–strain curve for cyclic loading. Number of cycles = ten times.

This alloy does not have superelasticity. The cyclic loading investigation was not performed on the test material because the expected effect of superelasticity did not occur after quenching and, accordingly, this post-annealing test will not provide any additional information.

### 3.7. Young’s Modulus

The Young’s modulus of the alloys after quenching from 600 °C and annealing at 400 °C for 1 h was determined using the nanoindentation method. Using the nanoindentation method on TiZrNb alloys allows us to determine Young’s modulus with high accuracy corresponding to the static test method with the use of an additional extensometer, but the method applied in this article has better performance. Young’s modulus determination during static tests on TiZrNb alloys without an extensometer shows consistently underestimated values and is not applied in this article.

**Figure 8:**
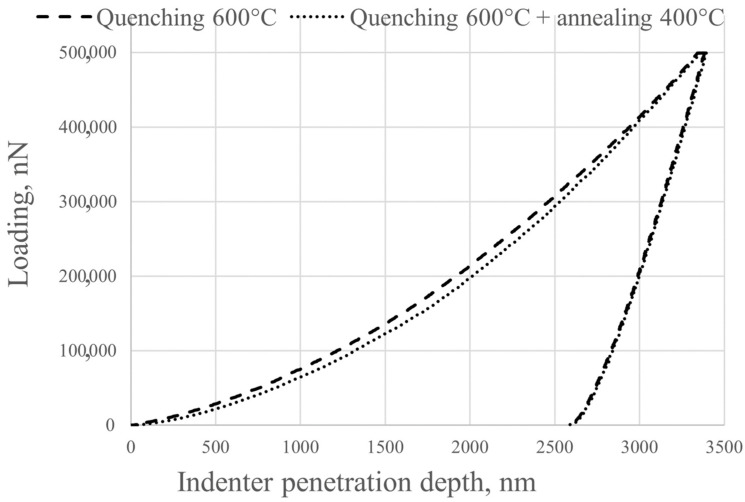
Diagram of alloy loading during indentation into the alloy.

An example of a loading diagram during indentation is shown in Figure 8. The results of the Young’s tests are presented in Table 7.

The observed decrease in Young’s modulus from 85 GPa to 81 GPa following low-temperature annealing, accompanied by the retention of the β-phase, may be attributed to microstructural alterations. The reduction in residual stresses due to quenching leads to a decrease in crystal lattice distortions, thereby lowering the local stiffness of the material. Atomic ordering, defined as short-distance order, or the redistribution of alloying elements within the β-matrix, serves to modify interatomic interactions, consequently affecting the material’s elastic properties. A decline in dislocation density during the annealing process weakens their contribution to elastic strength. Additionally, the potential formation of nano-sized clusters or local inhomogeneities, not detected using X-ray diffraction, can modify the deformation response. These processes, without affecting the phase composition, reduce the global stiffness of the material, which is manifested by a decrease in Young’s modulus during nanoindentation.

### 3.8. Fractography

**Figure 9:**
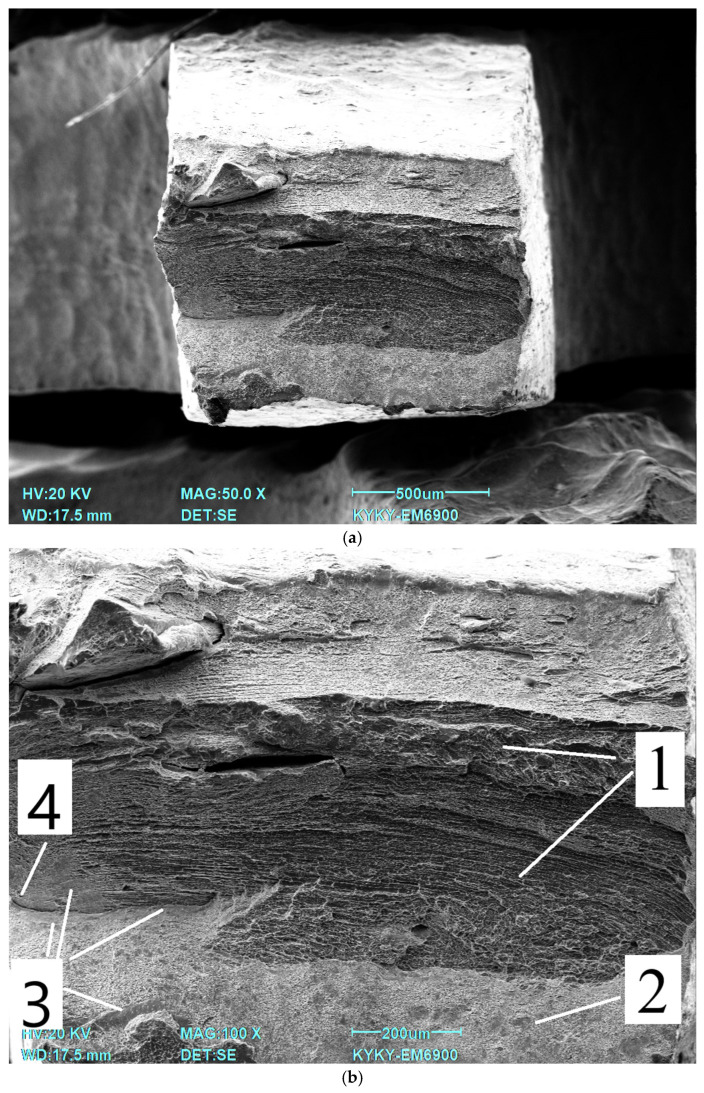

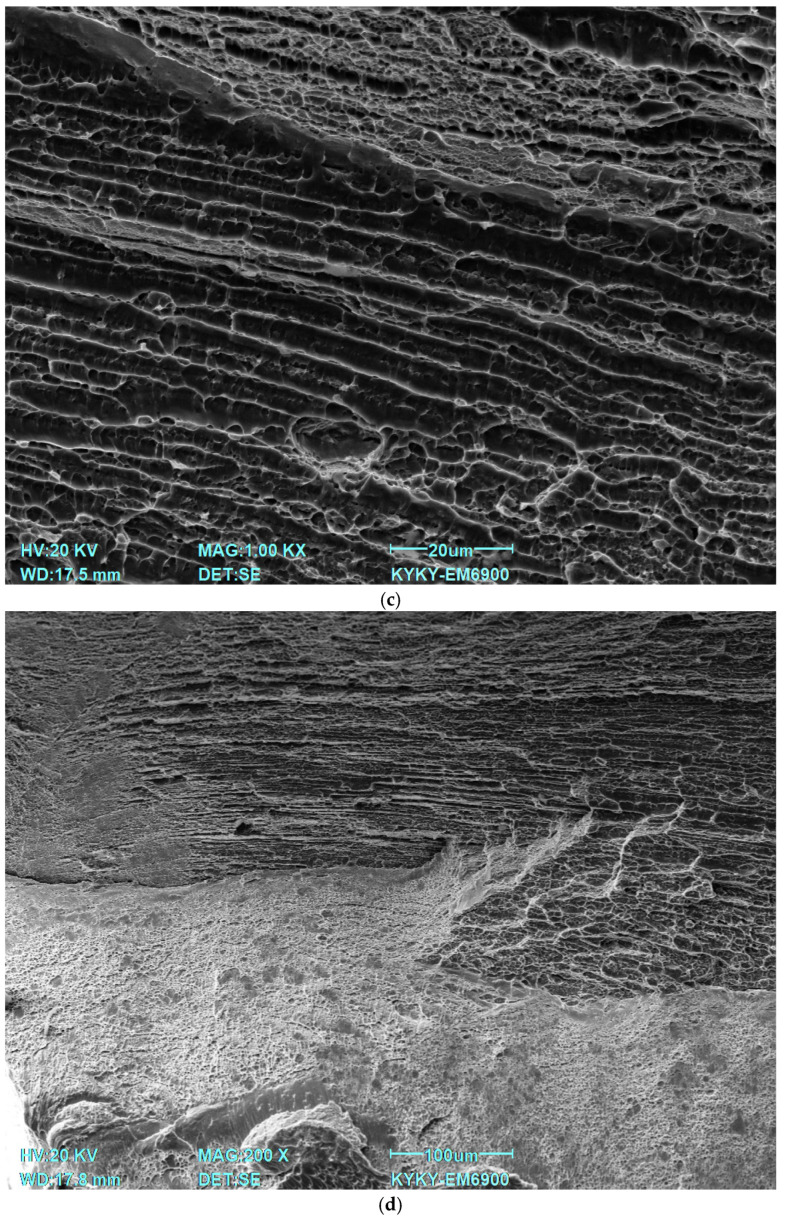
Fractography after quenching: (**a**) overall view; (**b**) fracture part; (**c**) central part; (**d**) zone with the presence of brittle fracture.

An image of the sample photo after quenching is shown in Figure 9: 1—ductile fracture zone with noticeable material flow; 2—ductile fracture zone with formation of a microporous surface; 3—brittle fracture zone; 4—location of fracture onset.

**Figure 10:**
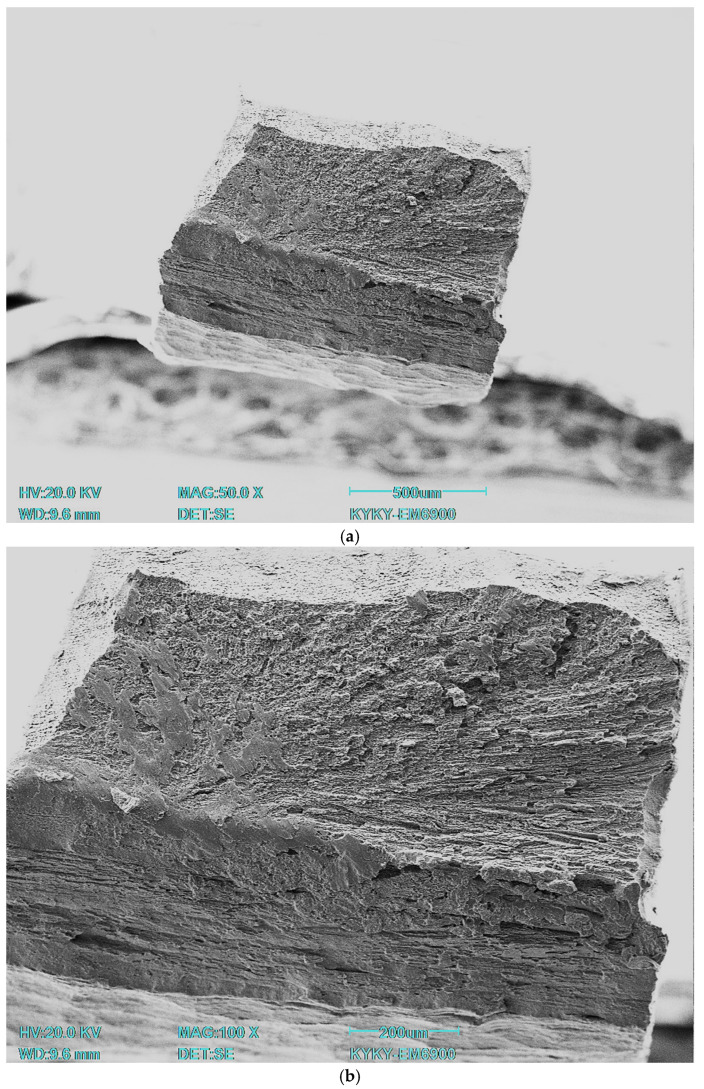

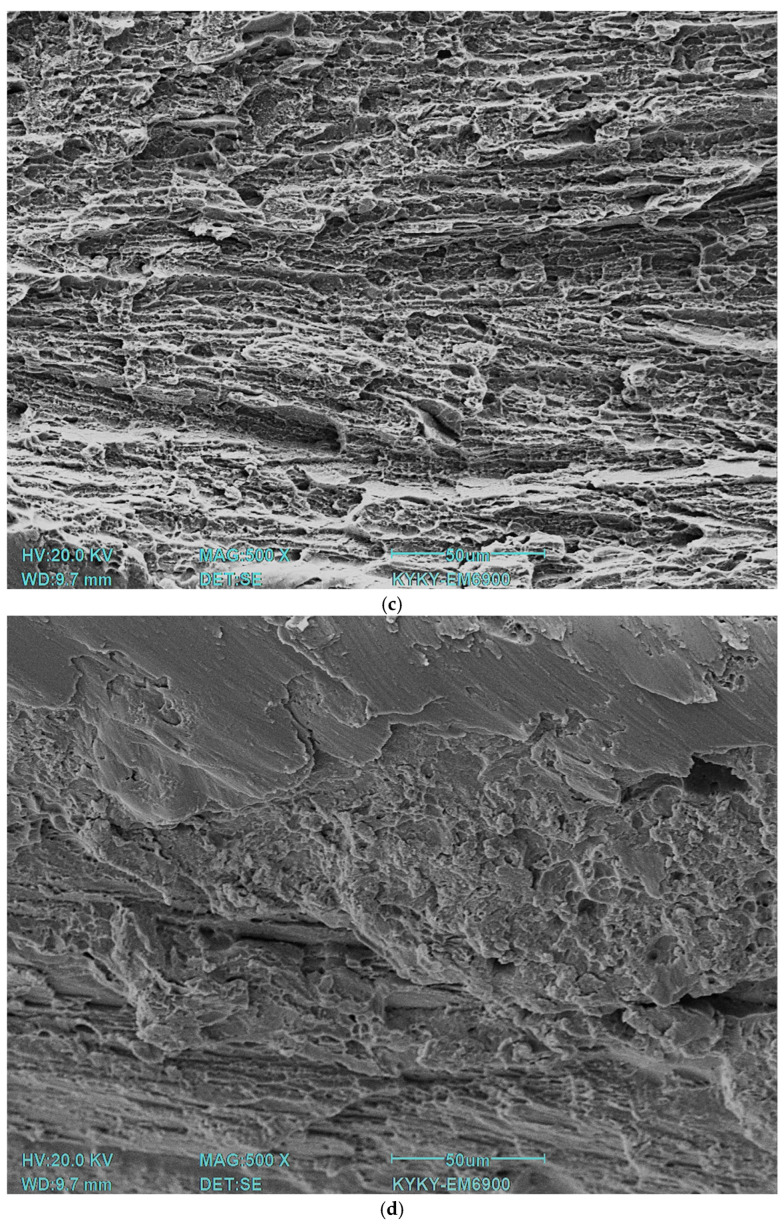
Fractography of Ti-38Zr-11Nb alloy after rolling, quenching, and annealing. (**a**) overall view; (**b**) view of fracture; (**c**) zone with viscous destruction; (**d**) zone with the presence of brittle fracture.

Figure 10 shows the fracture surface under static loading after rolling, quenching, and annealing.

SEM images show typical signs of tensile failure in the beta titanium alloy. A visual analysis reveals the following key features: the specimens underwent ductile fracture, resulting in the formation of a necking region; large areas with tensile structures are visible; areas of microporosity are present, which may be the result of the formation of plastic flow cells prior to fracture, characteristic of plastic failure; and areas with smooth surfaces are noticeable, which may indicate brittle local failure. Additionally, it is possible to note the presence of a layered structure, i.e., a central, more viscous area, and an area of increased plastic deformation on the outside, which was created during the production of the specimens, in which a high degree of deformation accumulates precisely on the sides of the specimen. On a large scale, it can be seen that the pits are not uniform in size, and some of them are connected by narrow bridges. This confirms the active plastic flow of the material. Small intercellular cracks are present in the specimen after quenching, which may indicate localized zones of brittle fracture at peak loads. Despite the general similarity in the behavior of specimens subjected to different types of heat treatment, it should be noted that in the annealed specimen the outer and inner regions are visually less distinct. This phenomenon can be explained by the stress relief due to annealing after quenching, where the specimen had internal stresses associated with a greater degree of deformation in the outer regions compared to the inner regions, as well as a more pronounced temperature gradient during quenching in the surface areas compared to the core.

### 3.9. Immersion Research

Before the samples were immersed in solutions, the masses and dimensions of the samples were measured. The values are shown in Table 8.

Table 9, Table 10 and Table 11 show the results of the determination of the contents of Al, Nb, V, Ti, and Zr in samples of immersion corrosion tests conducted in various buffer systems. The relative standard deviation (Sr) is 0.01–0.005 for elements from 1 to 20 mg/L and does not exceed 0.20 for elements from 1 to *n* × 10 micrograms/L.

The “<” sign indicates the detection limit. The desired element can be present in the range from 0 to the specified limit. Each element has its own limit, depending on its nature, the sensitivity of the analytical equipment, and the overall composition of the substance under study.

Therefore, it can be concluded that, during the three-week immersion testing period, the studied alloys and commercially pure titanium exhibited high corrosion resistance, in contrast to alloy VT6, where aluminum exhibited the highest yield and vanadium exhibited a slightly lower amount. The release of metals into the medium occurred almost uniformly, without gradual deceleration, which accompanies the formation of a protective passive film in titanium alloys after etching the defective areas from the sample surface. Concurrently, the concentration of the primary alloy element (titanium) remains below the detection limit of the device in all of the studied samples.

### 3.10. Corrosion Studies

**Figure 11:**
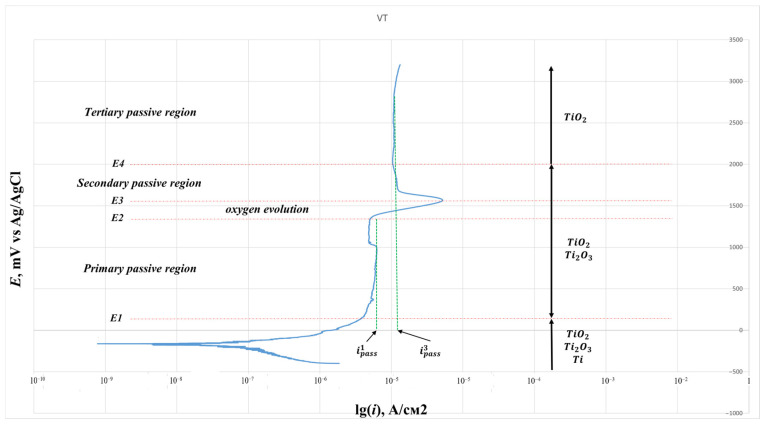
Passivation zones on the anode site and the types of oxide film in them.

Figure 11 shows the polarization curve for an industrial VT6 alloy with characteristic zones in the anode region.

**Figure 12:**
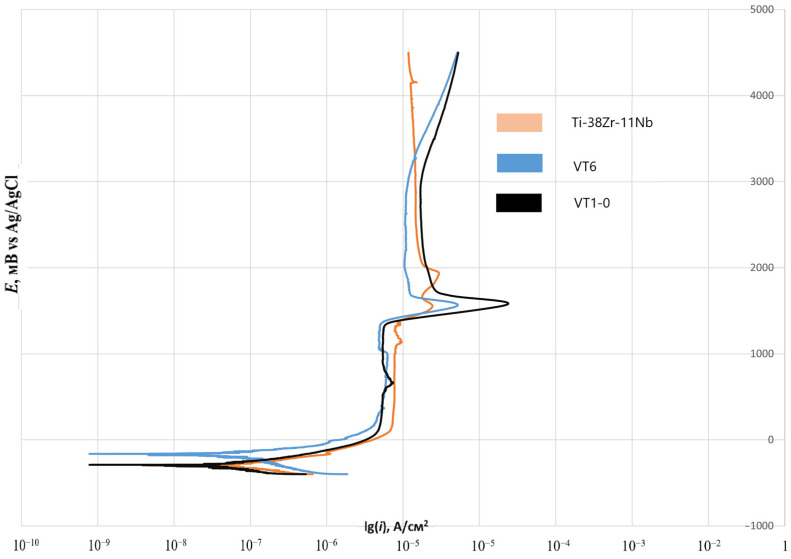
Polarization curves for industrial and experimental alloy in a model saline solution.

Figure 12 shows a summary graph of polarization curves in a model saline solution. The analysis shows that, for industrial alloys VT1-0, VT6, and Ti-38 Zr-11Nb, qualitative coordination of all sections on the anode branch is observed.

**Figure 13:**
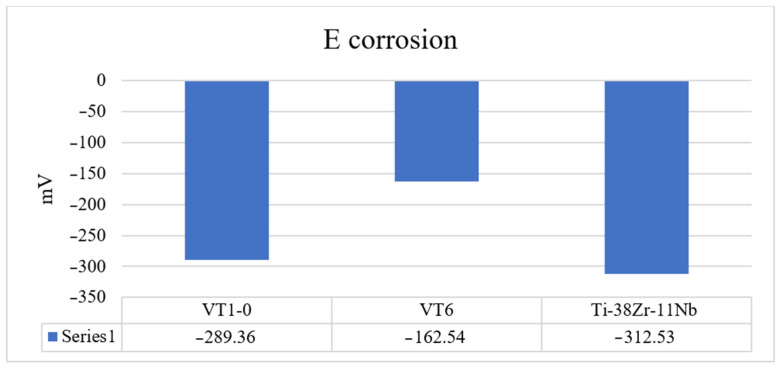
Corrosion potential in the model saline solution.

Figure 13 shows a bar chart of the corrosion potential for the alloys under study.

**Figure 14:**
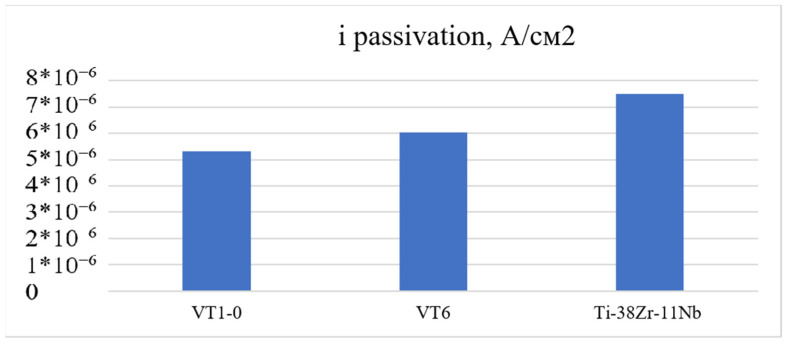
Current density at the first passivation site.

Figure 14 shows a bar chart for estimating the current density at the first passivation site. The quantitative (Table 12) assessment shows that the corrosion current density for the VT1-0 is the lowest, which indicates an earlier onset of passive film formation on the surface of the experimental alloy.

**Figure 15:**
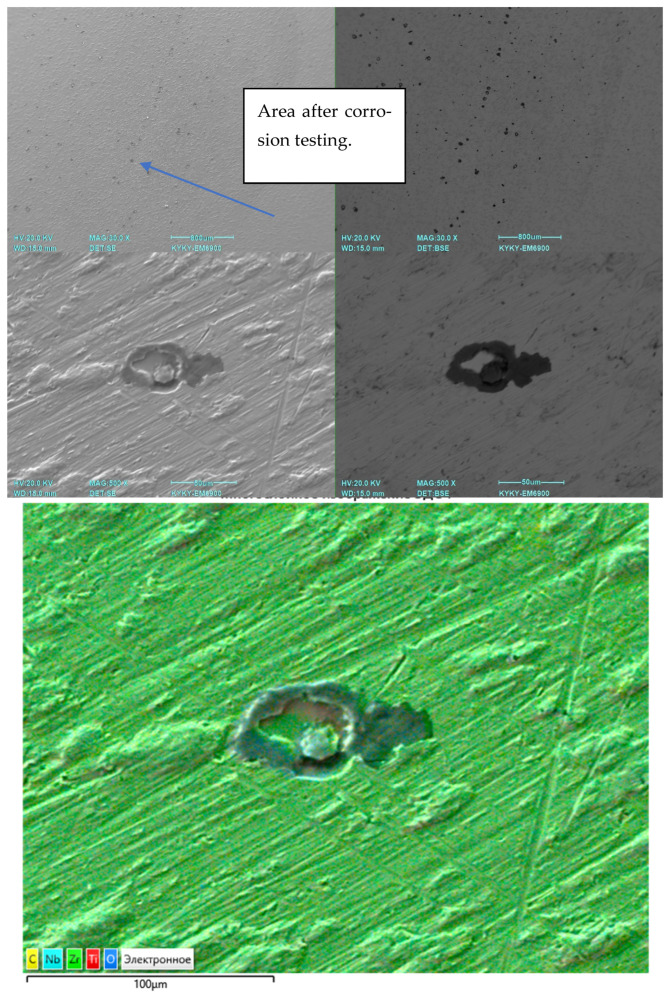

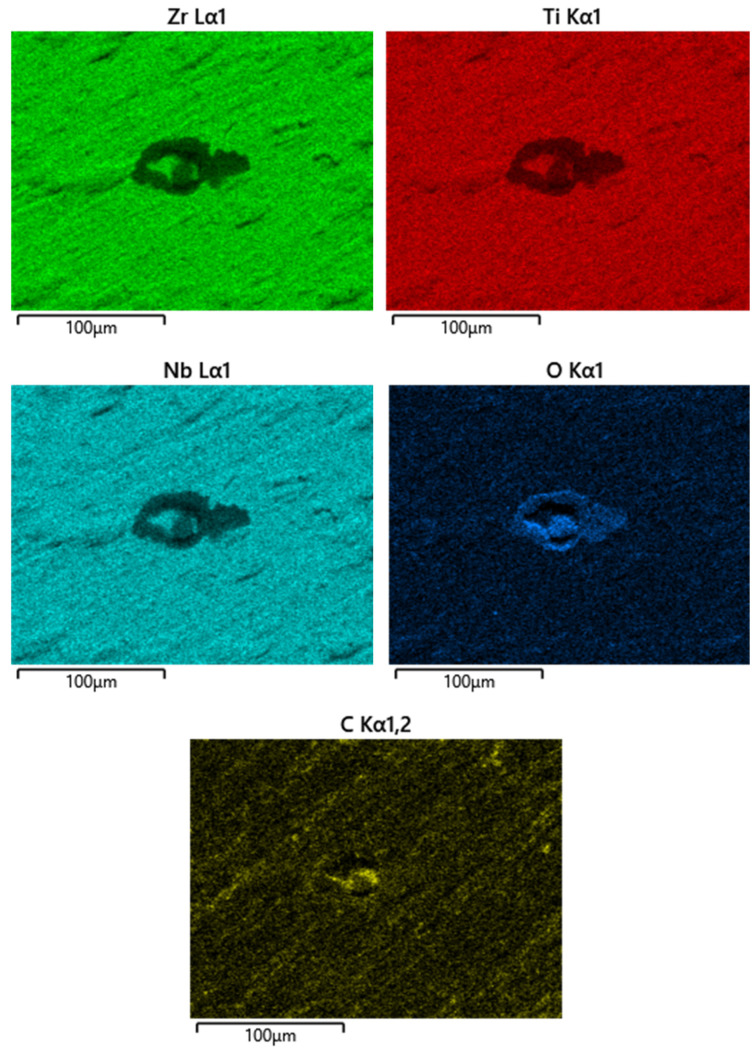
Pitting corrosion of Ti-38Zr-11Nb alloy after corrosion tests.

Figure 15 illustrates the pitting corrosion of the Ti-38Zr-11Nb alloy after corrosion tests.

The analysis of the corrosion behavior of industrial alloys VT1-0, VT6, and Ti-38 Zr-11Nb in saline solution (0.9% NaCl) indicates a qualitative agreement between the effect of chlorides on the corrosion film resistance on the surface of the alloys. The close values of the corrosion current density for all the studied alloys indicate comparable resistances to corrosion. Considering that VT1-0 and VT6 alloys have experimentally proven resistance to biological fluids in the human body, it can be concluded that Ti-38Zr-11Nb has potentially high corrosion resistance.

### 3.11. In Vitro Biocompatibility

In the course of the work, titanium samples with a diameter of no more than 0.5 cm were examined.

Before the tests, the surface polishing quality was evaluated to ensure the uniformity of the samples in the experiment, as it is known that roughness can significantly affect cell viability.

**Figure 16:**
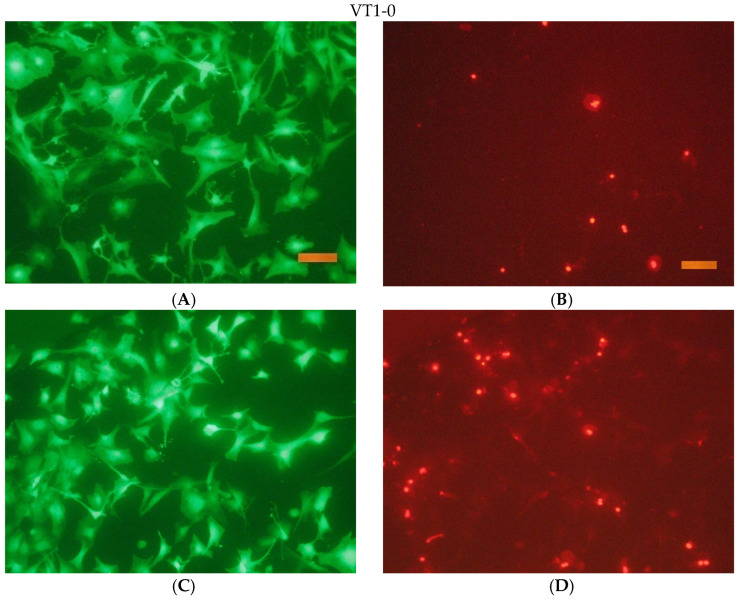
The appearance of cells during incubation on the surface of VT1-0 for 1 and 3 days. Coloring cells with paint Syto 9 (**A**,**C**); staining the nuclei of dead cells with paint PI (**B**,**D**). Size tag = 100 mkm.

**Figure 17:**
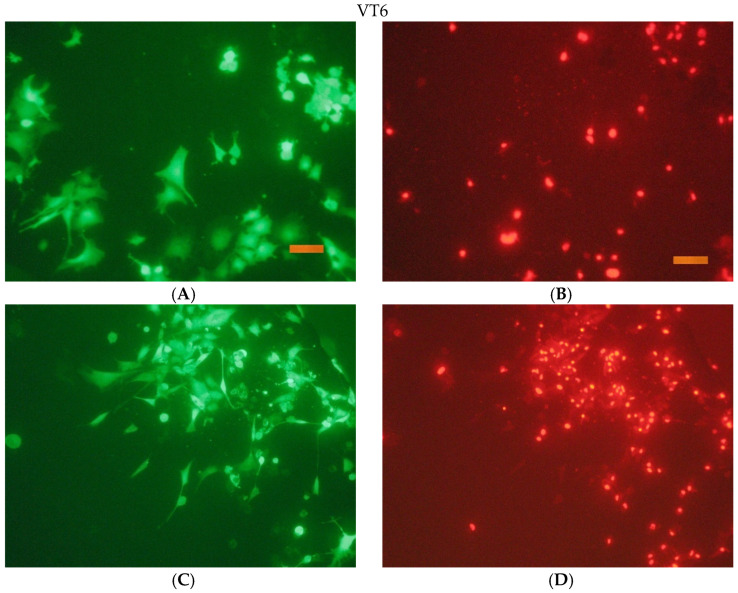
The appearance of cells during incubation on the surface of VT6 for 1 and 3 days. Coloring cells with paint Syto 9 (**A**,**C**); staining the nuclei of dead cells with paint PI (**B**,**D**). Size tag = 100 mkm.

**Figure 18:**
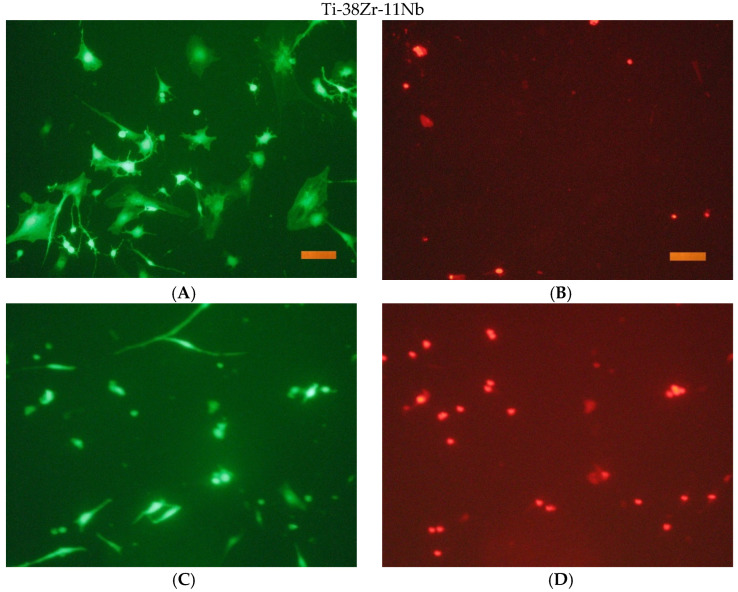
The appearance of cells during incubation on the surface of Ti-38Zr-11Nb for 1 and 3 days. Coloring cells with paint Syto 9 (**A**,**C**); staining the nuclei of dead cells with paint PI (**B**,**D**). Size tag = 100 mkm.

**Figure 19:**
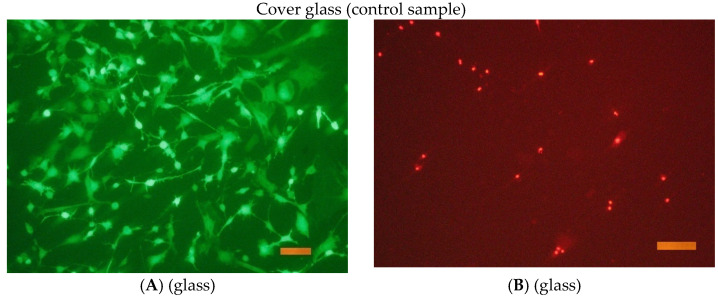

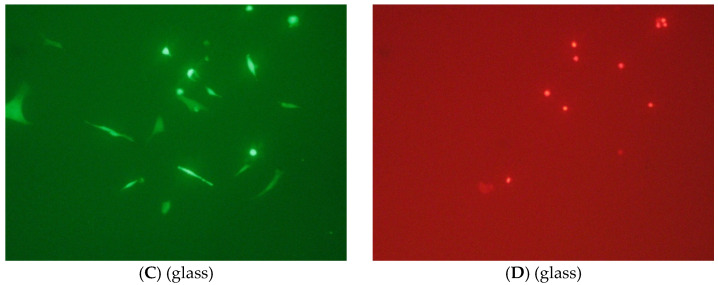
The appearance of cells during incubation on the surface of the cover glass for 1 and 3 days. Coloring cells with paint Syto 9 (**A**,**C**); staining the nuclei of dead cells with paint PI (**B**,**D**). Size tag = 100 mkm.

The results of a fluorescence microscopy study of the viability, adhesion, and proliferative activity of NCTC clone L929 during cultivation on the surface of titanium samples are shown in the following Figure 16, Figure 17, Figure 18 and Figure 19.

**Figure 20:**
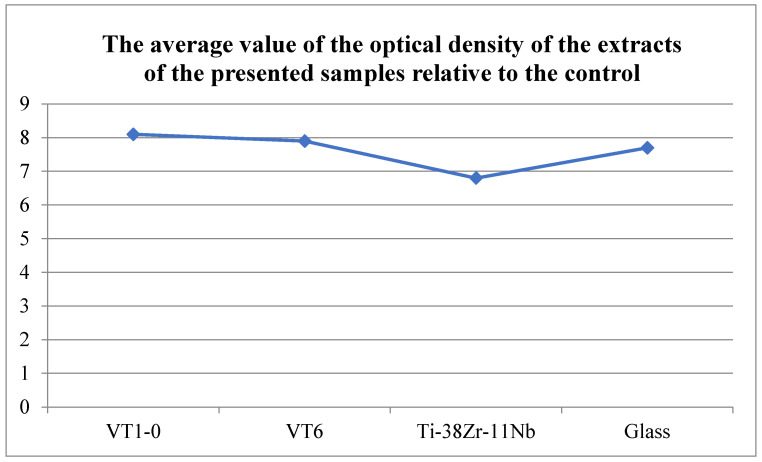
Optical density values of extracts from the presented materials during cultivation of the NCTC L929 line for 1 day according to the results of the MTT test.

**Figure 21:**
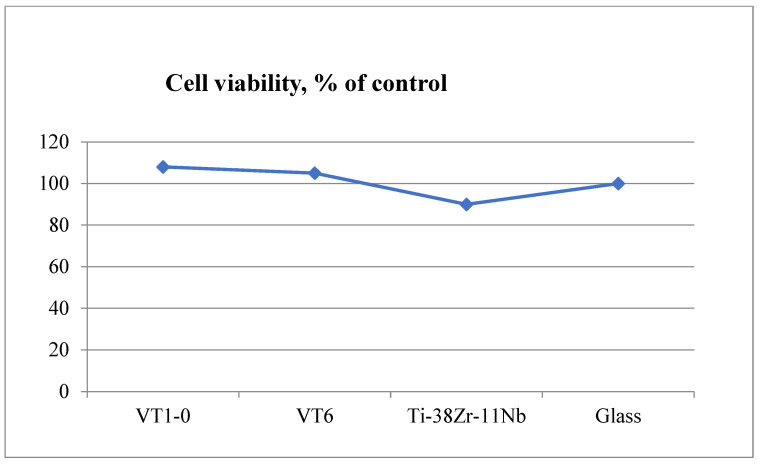
Viability of NCTC L929 cells during cultivation for 1 day in the presence of extracts from the presented materials of the MTT test.

The study of the metabolic activity of NCTC L929 cells in the presence of extracts from the studied materials was carried out using an MTT test. The results are shown in Figure 20 and Figure 21.

The conducted research has shown that these biomaterial samples are biocompatible and do not have cytotoxic effects on mammalian cells. No significant differences were found in the viability of NCTC L929 cells when cultured for 1 day in the presence of extracts from the presented materials. No significant (more than 15%) decrease in the metabolic activity of NCTC L929 cells was found when cultured for 1 day in the presence of extracts from the presented materials.

## 4. Conclusions

The beta phase in the Ti-38Zr-11Nb alloy was detected, as expected, after quenching, and was confirmed after low-temperature annealing. This confirms the long-term stability of the phase composition, which is important for the use of the material in implantology.

The uniform distribution of elements (Ti, Zr, Nb) in the alloy contributes to the homogeneity of its mechanical properties and biocompatibility, and the presence of zirconium in combination with niobium stabilizes the beta phase, improving the mechanical properties.

The low modulus of elasticity (reduction from 85 GPa to 81 GPa after annealing) brings the mechanical properties of the alloy closer to those of bone tissue, thereby minimizing the risk of stress shielding and improving the biomechanical compatibility of the implant. The enhancement in strength properties (an increase from 371 to 614 MPa) following quenching and annealing is linked to recrystallization, subgrain formation, the redistribution of residual stresses, and dislocation strengthening. The net effect of these changes is an enhancement in the mechanical stability of the implant.

The material’s plasticity diminished (relative strain decreased by 32%) due to the formation of an ordered structure that hinders dislocation movement and local microstructural changes.

Microstructural analysis revealed that the material shows signs of plastic failure with local zones of brittle failure, which requires the further optimization of composition and processing to increase durability and reliability.

The absence of α′ and ω phase formation (within the sensitivity of the device) after heat treatment confirms the stabilizing effect of niobium and zirconium on the beta phase, which is important for maintaining a low elastic modulus and biocompatibility of the material.

Analytical methods (energy dispersive spectrometry, X-ray phase analysis, nanohardness) confirmed the high homogeneity and stability of the material properties, making the Ti-38Zr-11Nb alloy a promising candidate for use in hip arthroplasty.

The comparison of the corrosion behavior of industrial materials with the investigated SPLPAV shows close values of corrosion current density, indicating comparable corrosion resistance in 0.9% NaCl medium.

During immersion tests over 3 weeks, the investigated alloy and commercially pure titanium showed high corrosion resistance, in contrast to the alloy BT6, where there was a yield of aluminum and somewhat less vanadium, and the release of metals into the medium occurred almost uniformly, without gradual retardation, which accompanied the formation of a protective passive film in the titanium alloys after the defects were removed from the surface of the sample.

The present study demonstrates that these commercial material samples, Ti-38Zr-13Nb and a glass control sample, are biocompatible and have no cytotoxic effects on mammalian cells.

## Figures and Tables

**Table 1:** Conversion of atomic percentage to mass percentage.

Chemical Element	Ti	Nb	Zr
Atomic mass (AMU)	47.87	92.91	91.22
Atomic Percent (at.%)	50	11	39
AMU * at.%	2393.5	1022.01	3557.58
Mass Percent (%)	34	15	51

* AMU–Atomic mass unit

**Table 2:** Expected phase composition after smelting [33].

Phase Composition	[Mo] eq
(α + β)-titanium alloy	3.3–10
Pseudo-β-titanium alloy	11.0–16.3
β-titanium alloy	16.9

**Table 3:** Impurity composition of the alloy.

Oxygen, Mass %	Nitrogen, Mass %	Hydrogen, Mass %	Carbon, Mass %	Sulfur, Mass %
0.02 ± 0.005	0.0059 ± 0.004	0.0064 ± 0.001	0.017 ± 0.005	0.0054 ± 0.0009

**Table 4:** Phase composition of Ti-38Zr-11Nb alloy plates.

State	Type of Phase	Type of Crystal Lattice	Contents, Volume %	Crystal Lattice Parameters
After quenching	β-Ti	type A2, cI2	100	a = 3.4 ± 0.01 Å
After quenching + annealing	β-Ti	type A2, cI2	100	a = 3.4 ± 0.01 Å

**Table 5:** Nanohardness of Ti-38Zr-11Nb plates.

Condition	Nanohardness, HV
After quenching	258 ± 7
After quenching + annealing	236 ± 10

**Table 6:** Results of the test of Ti-38Zr-11Nb plates.

State	Tensile, %	σ0.2, MPa	σ_B_, MPa
After quenching	12.2 ± 1.4	371 ± 35	529 ± 14
After quenching + annealing	9.3 ± 0.7	614 ± 20	628 ± 14

**Table 7:** Results of Young’s modulus values.

Composition	E, GPa
After quenching	85 ± 3
After quenching + annealing	81 ± 2

**Table 8:** Characteristics of the studied materials before immersion in the media.

Alloy	No	Weight, g	Length, mm	Height, mm	Width, mm
Rough surface					
VT1-0	1	0.1276	10.06	3.05	1.01
	2	0.1288	10.03	3.06	1
	3	0.1294	10	3.06	0.99
VT6	1	0.1062	9.6	2.99	0.85
	2	0.1099	9.99	3	0.85
	3	0.1089	9.97	3.01	0.9
Ti-38Zr-11Nb	1	0.3016	9.84	2.85	2.05
	2	0.2821	9.88	2.8	1.96
	3	0.2664	9.67	2.78	1.94
Polished surface					
Ti-38Zr-11Nb	1	0.2712	9.68	2.8	1.8
	2	0.2601	9.67	2.65	1.84
	3	0.2497	9.72	2.63	1.78

**Table 9:** The results of the determination of Al, Nb, V, Ti, and Zr in the sample of immersion corrosion tests on the 7th day.

Alloy	Al, ppm	V, ppm	Ti, ppm	Nb, ppm	Zr, ppm
Rough surface					
VT1-0	-	-	<0.025	-	-
VT6	0.3203	<0.025	<0.025	-	-
Ti-38Zr-11Nb	-	-	<0.025	<0.040	<0.025
Polished surface					
Ti-38Zr-11Nb	-	-	<0.025	<0.040	<0.025

**Table 10:** The results of the determination of Al, Nb, V, Ti, and Zr in the sample of immersion corrosion tests on the 14th day.

Alloy	Al, ppm	V, ppm	Ti, ppm	Nb, ppm	Zr, ppm
Rough surface					
VT1-0	-	-	<0.025	-	-
VT6	1.065	0.0396	<0.025	-	-
Ti-38Zr-11Nb	-	-	<0.025	<0.040	<0.025
Polished surface					
Ti-38Zr-11Nb	-	-	<0.025	<0.040	<0.025

**Table 11:** The results of the determination of Al, Nb, V, Ti, and Zr in the sample of immersion corrosion tests on the 21st day.

Alloy	Al, ppm	V, ppm	Ti, ppm	Nb, ppm	Zr, ppm
Rough surface					
VT1-0	-	-	<0.025	-	-
VT6	3.8284	0.1349	<0.025	-	-
Ti-38Zr-11Nb	-	-	<0.025	<0.040	<0.025
Polished surface					
Ti-38Zr-11Nb	-	-	<0.025	<0.040	<0.025

**Table 12:** Energy passivation of polarization curves for the studied alloys.

Alloy	E_corrosion_, mV	i_KOPP_, A/cm^2^	E1, mV	E2, mV	E3, mV	E4, mV	i_Passivation1_, A/cm^2^	i_Passivation3_, A/cm^2^
VT1-0	−289.36	3.45 × 10^−8^	100	1338	1578	2200	5.3 × 10^−6^	1.76 × 10^−5^
VT6	−162.54	4.33 × 10^−8^	162	1350	1565	2000	6.02 × 10^−6^	1.07 × 10^−5^
Ti-38Zr-11Nb	−296.6	5.32 × 10^−8^	100	1360	1545	2100	7.6 × 10^−6^	1.46 × 10^−5^

## Data Availability

The original contributions presented in this study are included in the article. Further inquiries can be directed to the corresponding author.
